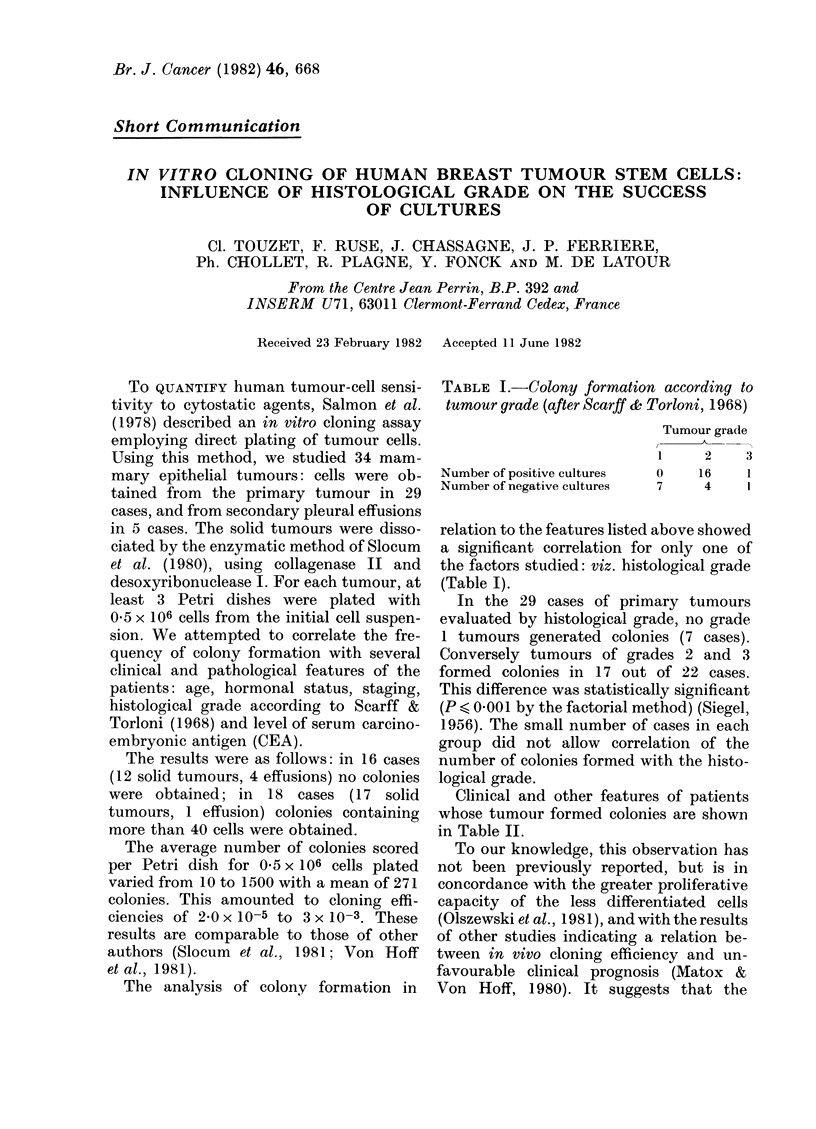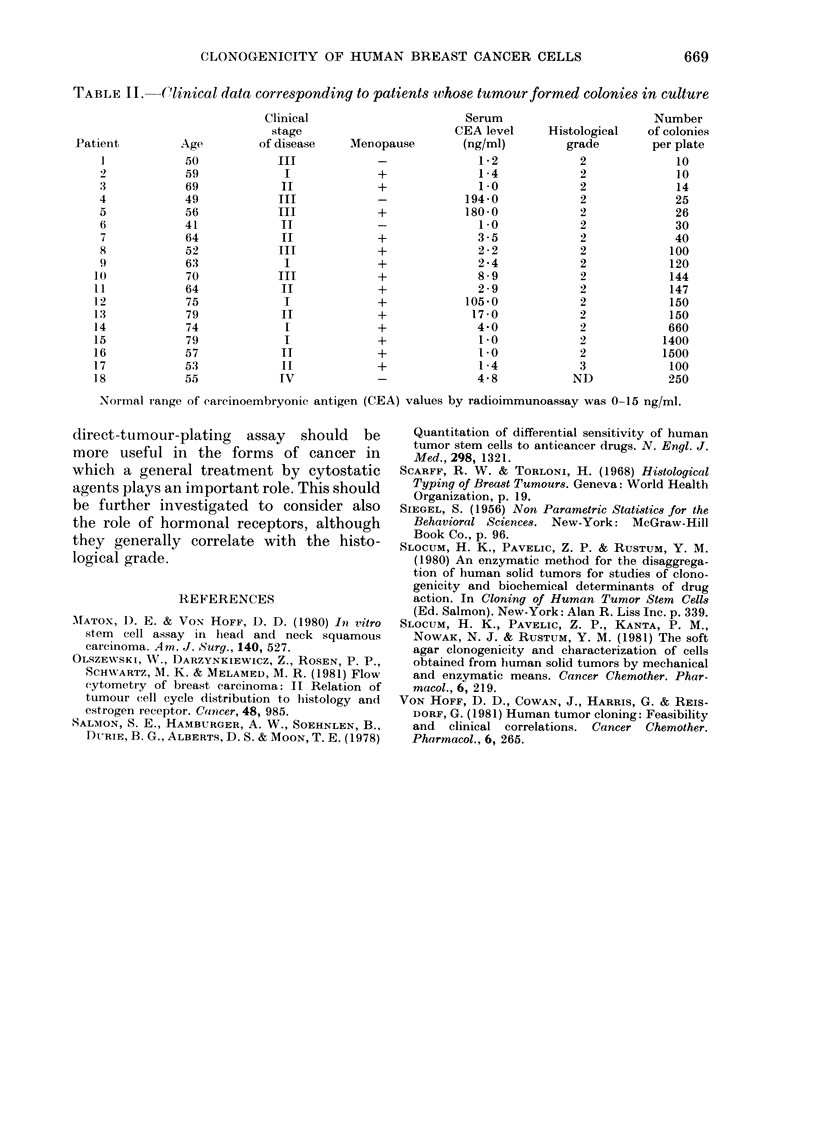# In vitro cloning of human breast tumour stem cells: influence of histological grade on the success of cultures.

**DOI:** 10.1038/bjc.1982.252

**Published:** 1982-10

**Authors:** C. Touzet, F. Ruse, J. Chassagne, J. P. Ferriere, P. Chollet, R. Plagne, Y. Fonck, M. de Latour


*Br. J. Cancer* (1982) **46**, 668

***Short Communication***

### *IN VITRO* CLONING OF HUMAN BREAST TUMOUR STEM CELLS: INFLUENCE OF HISTOLOGICAL GRADE ON THE SUCCESS OF CULTURES

Cl. TOUZET, F. RUSE, J. CHASSAGNE, J. P. FERRIERE, Ph. CHOLLET, R. PLAGNE, Y. FONCK AND M. DE LATOUR

*From the Centre Jean Perrin, B.P.* 392 *and INSERM U*71, 63011 *Clermont-Ferrand Cedex, France*

Received 23 February 1982 Accepted 11 June 1982

To QUANTIFY human tumour-cell sensitivity to cytostatic agents, Salmon *et al.* (1978) described an *in vitro* cloning assay employing direct plating of tumour cells. Using this method, we studied 34 mammary epithelial tumours: cells were obtained from the primary tumour in 29 cases, and from secondary pleural effusions in 5 cases. The solid tumours were dissociated by the enzymatic method of Slocum *et al.* (1980), using collagenase II and desoxyribonuclease I. For each tumour, at least 3 Petri dishes were plated with $0{\cdot}5 \times 10^6$ cells from the initial cell suspension. We attempted to correlate the frequency of colony formation with several clinical and pathological features of the patients: age, hormonal status, staging, histological grade according to Scarff & Torloni (1968) and level of serum carcinoembryonic antigen (CEA).

The results were as follows: in 16 cases (12 solid tumours, 4 effusions) no colonies were obtained; in 18 cases (17 solid tumours, 1 effusion) colonies containing more than 40 cells were obtained.

The average number of colonies scored per Petri dish for $0{\cdot}5 \times 10^6$ cells plated varied from 10 to 1500 with a mean of 271 colonies. This amounted to cloning efficiencies of $2{\cdot}0 \times 10^{-5}$ to $3 \times 10^{-3}$. These results are comparable to those of other authors (Slocum *et al.*, 1981; Von Hoff *et al.*, 1981).

The analysis of colony formation in relation to the features listed above showed a significant correlation for only one of the factors studied: *viz.* histological grade (Table I).

TABLE I.—*Colony formation according to tumour grade* (*after Scarff & Torloni*, 1968)

| | Tumour grade | | |
|---|---|---|---|
| | 1 | 2 | 3 |
| Number of positive cultures | 0 | 16 | 1 |
| Number of negative cultures | 7 | 4 | 1 |

In the 29 cases of primary tumours evaluated by histological grade, no grade 1 tumours generated colonies (7 cases). Conversely tumours of grades 2 and 3 formed colonies in 17 out of 22 cases. This difference was statistically significant ($P \leqslant 0{\cdot}001$ by the factorial method) (Siegel, 1956). The small number of cases in each group did not allow correlation of the number of colonies formed with the histological grade.

Clinical and other features of patients whose tumour formed colonies are shown in Table II.

To our knowledge, this observation has not been previously reported, but is in concordance with the greater proliferative capacity of the less differentiated cells (Olszewski *et al.*, 1981), and with the results of other studies indicating a relation between *in vivo* cloning efficiency and unfavourable clinical prognosis (Matox & Von Hoff, 1980). It suggests that the

TABLE II.—*Clinical data corresponding to patients whose tumour formed colonies in culture*

| Patient | Age | Clinical stage of disease | Menopause | Serum CEA level (ng/ml) | Histological grade | Number of colonies per plate |
|---|---|---|---|---|---|---|
| 1 | 50 | III | − | 1·2 | 2 | 10 |
| 2 | 59 | I | + | 1·4 | 2 | 10 |
| 3 | 69 | II | + | 1·0 | 2 | 14 |
| 4 | 49 | III | − | 194·0 | 2 | 25 |
| 5 | 56 | III | + | 180·0 | 2 | 26 |
| 6 | 41 | II | − | 1·0 | 2 | 30 |
| 7 | 64 | II | + | 3·5 | 2 | 40 |
| 8 | 52 | III | + | 2·2 | 2 | 100 |
| 9 | 63 | I | + | 2·4 | 2 | 120 |
| 10 | 70 | III | + | 8·9 | 2 | 144 |
| 11 | 64 | II | + | 2·9 | 2 | 147 |
| 12 | 75 | I | + | 105·0 | 2 | 150 |
| 13 | 79 | II | + | 17·0 | 2 | 150 |
| 14 | 74 | I | + | 4·0 | 2 | 660 |
| 15 | 79 | I | + | 1·0 | 2 | 1400 |
| 16 | 57 | II | + | 1·0 | 2 | 1500 |
| 17 | 53 | II | + | 1·4 | 3 | 100 |
| 18 | 55 | IV | − | 4·8 | ND | 250 |

Normal range of carcinoembryonic antigen (CEA) values by radioimmunoassay was 0–15 ng/ml.

direct-tumour-plating assay should be more useful in the forms of cancer in which a general treatment by cytostatic agents plays an important role. This should be further investigated to consider also the role of hormonal receptors, although they generally correlate with the histological grade.

### REFERENCES

MATOX, D. E. & VON HOFF, D. D. (1980) *In vitro* stem cell assay in head and neck squamous carcinoma. *Am. J. Surg.*, **140**, 527.

OLSZEWSKI, W., DARZYNKIEWICZ, Z., ROSEN, P. P., SCHWARTZ, M. K. & MELAMED, M. R. (1981) Flow cytometry of breast carcinoma: II Relation of tumour cell cycle distribution to histology and estrogen receptor. *Cancer*, **48**, 985.

SALMON, S. E., HAMBURGER, A. W., SOEHNLEN, B., DURIE, B. G., ALBERTS, D. S. & MOON, T. E. (1978) Quantitation of differential sensitivity of human tumor stem cells to anticancer drugs. *N. Engl. J. Med.*, **298**, 1321.

SCARFF, R. W. & TORLONI, H. (1968) *Histological Typing of Breast Tumours.* Geneva: World Health Organization, p. 19.

SIEGEL, S. (1956) *Non Parametric Statistics for the Behavioral Sciences.* New-York: McGraw-Hill Book Co., p. 96.

SLOCUM, H. K., PAVELIC, Z. P. & RUSTUM, Y. M. (1980) An enzymatic method for the disaggregation of human solid tumors for studies of clonogenicity and biochemical determinants of drug action. In *Cloning of Human Tumor Stem Cells* (Ed. Salmon). New-York: Alan R. Liss Inc. p. 339.

SLOCUM, H. K., PAVELIC, Z. P., KANTA, P. M., NOWAK, N. J. & RUSTUM, Y. M. (1981) The soft agar clonogenicity and characterization of cells obtained from human solid tumors by mechanical and enzymatic means. *Cancer Chemother. Pharmacol.*, **6**, 219.

VON HOFF, D. D., COWAN, J., HARRIS, G. & REISDORF, G. (1981) Human tumor cloning: Feasibility and clinical correlations. *Cancer Chemother. Pharmacol.*, **6**, 265.